# Ischemic Stricture of the Terminal Ileum in the Setting of Superior Mesenteric Artery Stenting: A Case Report

**DOI:** 10.7759/cureus.102237

**Published:** 2026-01-24

**Authors:** Anas E Ahmed, Othman D Alamri, Mohamed F Alqahtani, Salman A Ageel, Ibrahim M Aldandan

**Affiliations:** 1 Community Medicine, Jazan University, Jazan, SAU; 2 College of Medicine, King Faisal University, Al-Ahsa, SAU; 3 College of Medicine, King Saud University, Riyadh, SAU; 4 College of Medicine, Alfaisal University, Riyadh, SAU; 5 Faculty of Medicine, King Faisal University, Al-Ahsa, SAU

**Keywords:** abdominal pain, chronic intestinal ischemia, chronic mesenteric ischemia, endovascular stenting, ileal narrowing, ischemic small bowel stricture, mesenteric revascularization, superior mesenteric artery stenosis, terminal ileum stricture

## Abstract

Chronic mesenteric ischemia is an infrequent but potentially debilitating condition that can result in irreversible intestinal injury when diagnosis or treatment is delayed. Although revascularization can restore mesenteric blood flow and improve symptoms, structural bowel damage acquired during prolonged hypoperfusion may persist or manifest later. Chronic ischemic strictures of the small intestine are rare and diagnostically challenging, particularly when involving the terminal ileum, where they may mimic inflammatory, infectious, or neoplastic disorders. We report the case of a 55-year-old woman with multiple cardiovascular risk factors who presented with progressive postprandial abdominal pain, weight loss, and early satiety. Initial imaging revealed high-grade stenosis of the superior mesenteric artery, which was confirmed on angiography and successfully treated with endovascular stent placement. Despite partial symptomatic improvement, the patient re-presented weeks later with acute right lower quadrant abdominal pain. Repeat imaging demonstrated segmental narrowing and inflammatory changes of the terminal ileum, while the mesenteric stent remained patent. Further evaluation with a barium follow-through study confirmed a fixed, short-segment ileal stricture consistent with chronic disease. After exclusion of alternative etiologies, a diagnosis of chronic ischemic stricture of the terminal ileum was established, likely related to longstanding mesenteric hypoperfusion before revascularization. The patient was managed conservatively with clinical improvement and remains under close follow-up. This case emphasizes that restoration of mesenteric arterial flow does not necessarily prevent delayed ischemic complications of the bowel. Persistent or new gastrointestinal symptoms following revascularization should prompt evaluation for chronic ischemic strictures, particularly in patients with significant vascular risk factors, to avoid misdiagnosis and delays in appropriate management.

## Introduction

Chronic mesenteric ischemia is an uncommon but clinically significant condition resulting from prolonged inadequate blood flow to the intestines, most frequently due to atherosclerotic disease affecting the mesenteric vessels [1]. The superior mesenteric artery (SMA) is most commonly involved, given its dominant role in supplying the small intestine and proximal colon [1,2]. While chronic mesenteric ischemia classically presents with postprandial abdominal pain, weight loss, and food fear, delayed or incomplete diagnosis can lead to irreversible ischemic injury of the bowel wall [1-3]. Over time, repeated episodes of hypoperfusion may result in fibrosis, scarring, and the formation of fixed intestinal strictures, a rare but important late complication [1,3].

Ischemic strictures of the small intestine are particularly uncommon and pose a diagnostic challenge due to their nonspecific clinical and radiologic features, often mimicking inflammatory bowel disease, infectious enteritis, or neoplasia [1]. The terminal ileum is an unusual site for chronic ischemic strictures, as it is typically supplied by collateral circulation; however, severe or prolonged vascular insufficiency may overcome these protective mechanisms [2,3]. Advances in endovascular therapy, including angioplasty and stenting of mesenteric arteries, have improved outcomes in chronic mesenteric ischemia, but bowel damage sustained before revascularization may still manifest later as stricturing disease [1-4]. Reporting such cases is essential to enhance clinical awareness, highlight diagnostic pitfalls, and emphasize the importance of considering ischemic etiologies in patients with ileal strictures and significant vascular risk factors.

## Case presentation

A 55-year-old woman was admitted to our institution with a history of recurrent abdominal symptoms that evolved over several months. The patient had significant cardiovascular risk factors, including long-standing hypertension, type 2 diabetes mellitus, dyslipidemia, and a history of smoking. There was no prior history of inflammatory bowel disease, abdominal surgery, radiation exposure, or known hypercoagulable disorders. The patient initially reported postprandial epigastric and periumbilical pain associated with early satiety and unintentional weight loss. These symptoms were progressive and led to multiple outpatient evaluations before definitive imaging was obtained.

On initial presentation, the patient was hemodynamically stable. Physical examination revealed a soft but mildly tender abdomen, most pronounced in the periumbilical region, without guarding or rebound tenderness. Bowel sounds were present but hypoactive. There was no abdominal distension, organomegaly, or palpable masses. Rectal examination was unremarkable, with no evidence of melena or hematochezia. Peripheral pulses were diminished but palpable, and no abdominal bruits were appreciated.

**Figure 1:**
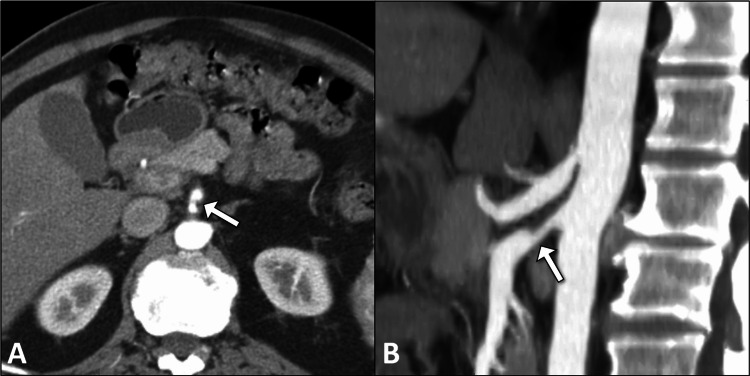
Axial and sagittal contrast-enhanced CT images showing SMA narrowing Axial (A) and sagittal (B) contrast-enhanced abdominal CT images demonstrate focal narrowing of the SMA (arrows), suggestive of chronic mesenteric ischemia. No evidence of thrombosis or aneurysmal dilation is seen. CT: computed tomography; SMA: superior mesenteric artery.

Initial laboratory investigations showed mild normocytic anemia, with hemoglobin slightly below baseline, and marginally elevated inflammatory markers. White blood cell count, liver function tests, renal function, serum electrolytes, lactate, and coagulation profile were within normal limits (Table 1). Given the chronicity of symptoms and risk factors for mesenteric ischemia, a contrast-enhanced computed tomography (CT) scan of the abdomen was performed. This demonstrated significant focal stenosis of the SMA with reduced distal opacification, raising concern for chronic mesenteric ischemia (Figure 1).

**Table 1:** Summary of laboratory investigations at presentation and during diagnostic workup This table summarizes the hematological, biochemical, immunological, and urinary laboratory findings obtained during the patient’s initial evaluation. Reference ranges correspond to standard adult values.

Laboratory Test	Value	Unit	Reference Range
Hemoglobin	10.8	g/dL	12.0–15.5
Hematocrit	33	%	36–46
Red Blood Cell Count	3.7	×10⁶/µL	4.0–5.2
Mean Corpuscular Volume	89	fL	80–96
Mean Corpuscular Hemoglobin	29	pg	27–33
Mean Corpuscular Hemoglobin Concentration	33	g/dL	32–36
Red Cell Distribution Width	14.8	%	11.5–14.5
White Blood Cell Count	12.6	×10³/µL	4.0–10.0
Neutrophils	78	%	40–75
Lymphocytes	15	%	20–45
Monocytes	5	%	2–10
Eosinophils	1	%	0–6
Basophils	1	%	0–2
Platelet Count	380	×10³/µL	150–400
C-reactive Protein	38	mg/L	<5
Erythrocyte Sedimentation Rate	42	mm/hr	<20
Blood Urea Nitrogen	18	mg/dL	7–20
Creatinine	0.9	mg/dL	0.6–1.2
Sodium	138	mmol/L	135–145
Potassium	4.2	mmol/L	3.5–5.1
Chloride	102	mmol/L	98–107
Bicarbonate	24	mmol/L	22–29
Calcium	9.1	mg/dL	8.6–10.2
Magnesium	1.9	mg/dL	1.7–2.2
Aspartate Aminotransferase	22	U/L	10–40
Alanine Aminotransferase	25	U/L	7–56
Alkaline Phosphatase	88	U/L	44–147
Total Bilirubin	0.6	mg/dL	0.1–1.2
Albumin	3.2	g/dL	3.5–5.0
Total Protein	6.1	g/dL	6.0–8.3
Prothrombin Time	12.8	seconds	11–14
International Normalized Ratio	1.1	-	0.8–1.2
Activated Partial Thromboplastin Time	31	seconds	25–35
Serum Lactate	1.6	mmol/L	0.5–2.2
Random Blood Glucose	168	mg/dL	70–140
HbA1c	7.8	%	<5.7
Ferritin	38	ng/mL	20–200
Serum Iron	42	µg/dL	50–170
Total Iron-Binding Capacity	410	µg/dL	250–450
Transferrin Saturation	10	%	20–50

**Figure 2:**
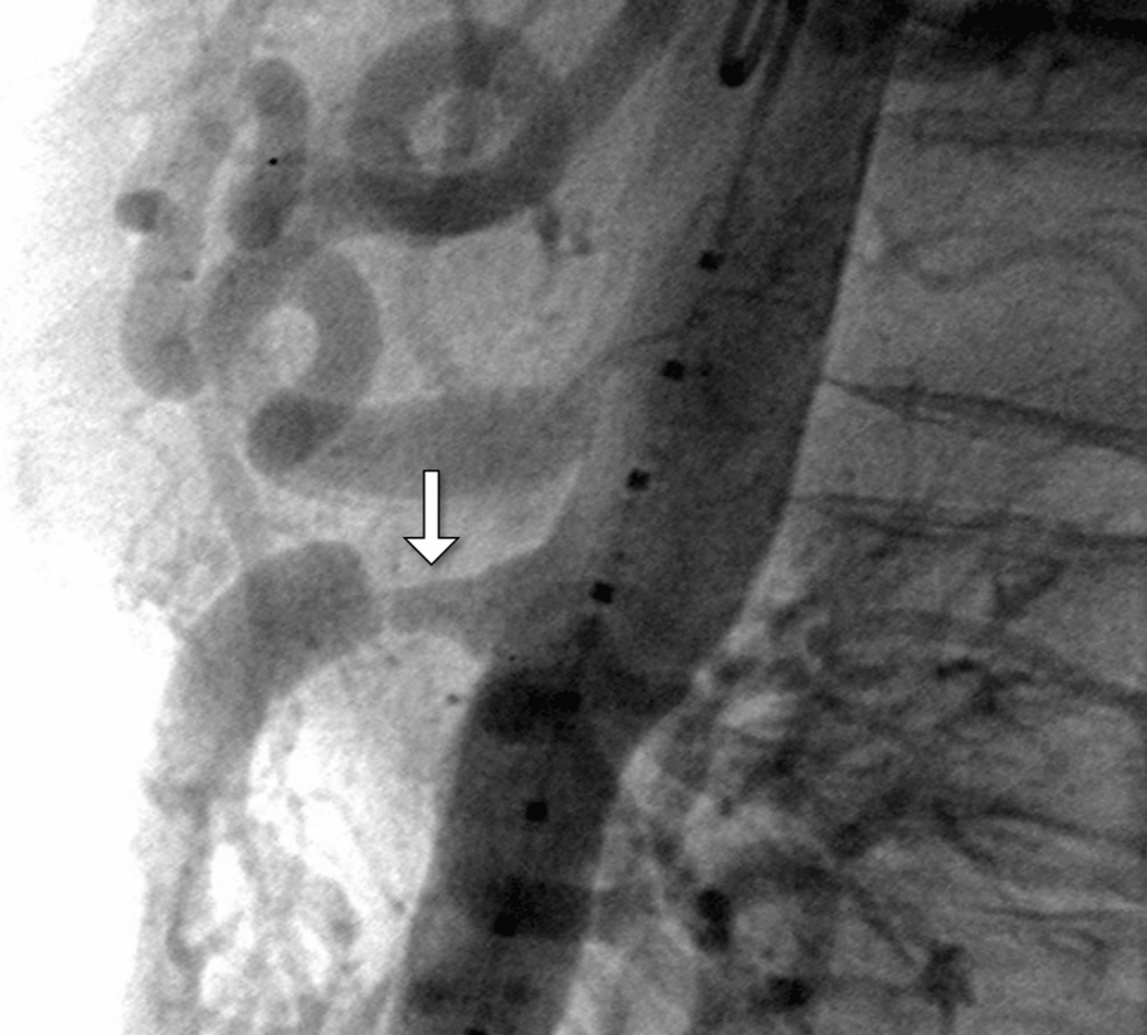
DSA confirming SMA stenosis. DSA of the mesenteric vessels reveals severe focal narrowing of the SMA (arrow) with preserved distal flow. The findings correlate with the CT evidence of mesenteric ischemia. DSA: digital subtraction angiography; SMA: superior mesenteric artery.

**Figure 3:**
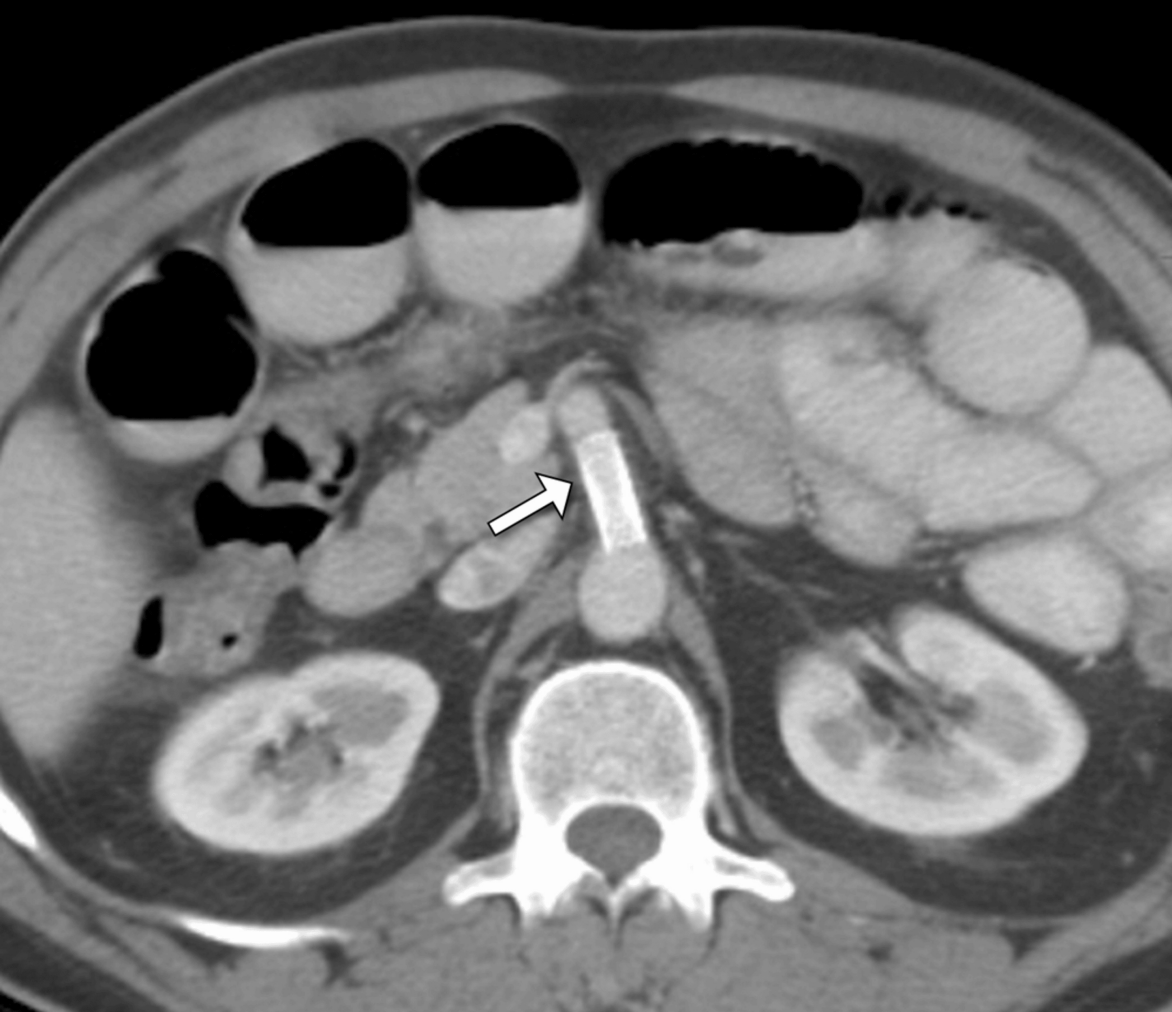
Axial CT demonstrating SMA stent placement Axial contrast-enhanced CT shows a metallic stent deployed within the SMA (arrow), with restored luminal caliber. There is no evidence of stent migration or perivascular complication. CT: computed tomography; SMA: superior mesenteric artery.

To further characterize the vascular pathology, digital subtraction angiography was undertaken, confirming high-grade stenosis of the proximal SMA (Figure 2). In view of the patient’s symptoms and imaging findings, a multidisciplinary decision was made to proceed with endovascular intervention. Percutaneous transluminal angioplasty with stent placement across the stenotic segment of the SMA was successfully performed without immediate complications. A post-procedure computed tomography angiogram confirmed appropriate positioning and patency of the SMA stent with restoration of distal flow (Figure 3).

Following the intervention, the patient experienced partial symptomatic relief and was discharged on dual antiplatelet therapy with close outpatient follow-up. However, several weeks later, the patient re-presented to the emergency department with an acute onset of abdominal pain localized to the right lower quadrant, associated with nausea and decreased oral intake. There was no vomiting or overt gastrointestinal bleeding. On examination, the patient appeared uncomfortable but remained hemodynamically stable. Abdominal examination revealed localized tenderness in the right iliac fossa with mild voluntary guarding but no peritoneal signs.

**Figure 4:**
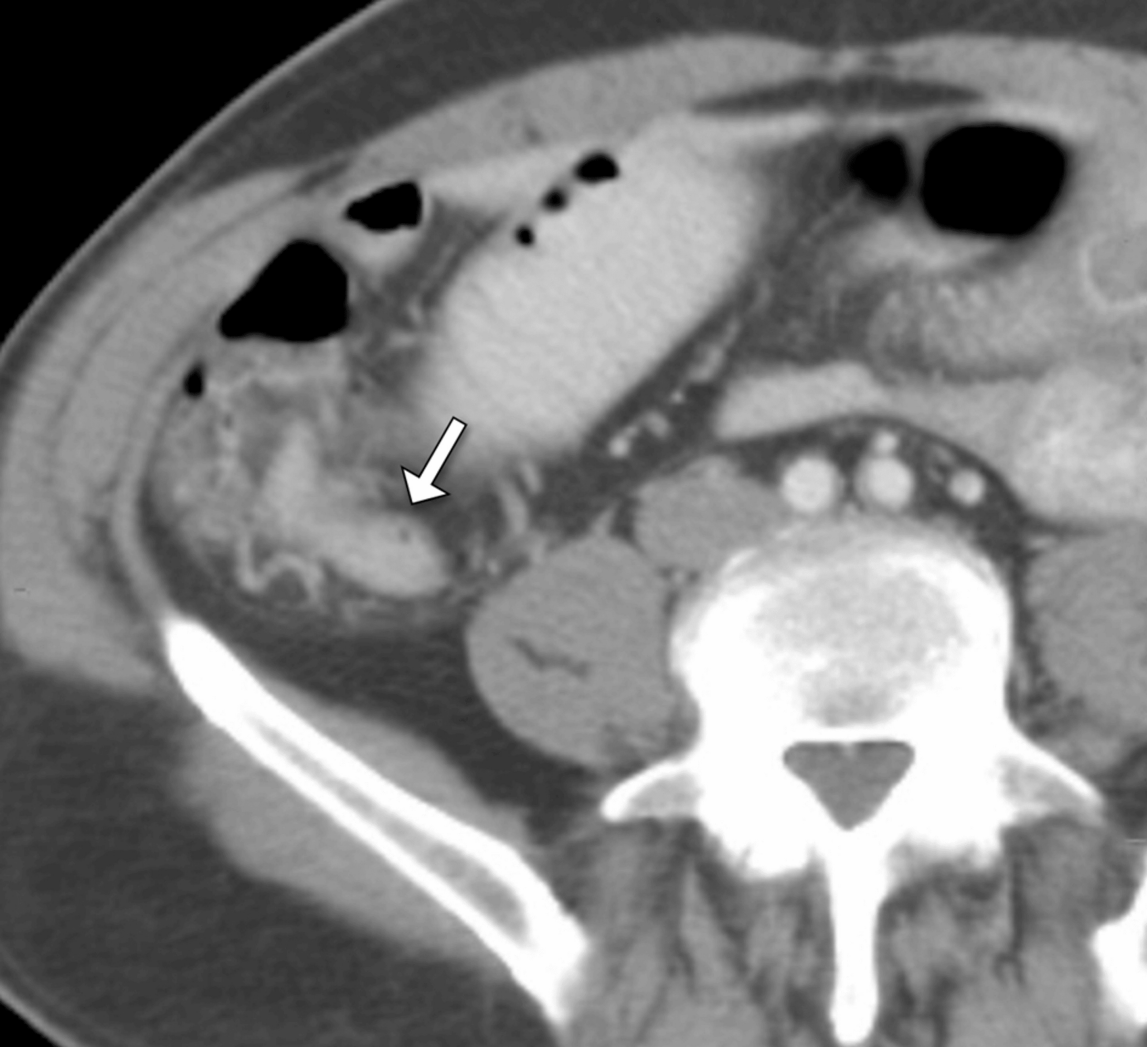
Axial CT showing terminal ileum thickening and stricture formation Axial contrast-enhanced CT of the abdomen demonstrates circumferential wall thickening and luminal narrowing of the terminal ileum (arrow), consistent with chronic ischemic stricture. No upstream dilatation of the small bowel is noted. CT: computed tomography.

A repeat contrast-enhanced computed tomography scan of the abdomen and pelvis demonstrated segmental circumferential wall thickening and luminal narrowing of the terminal ileum with associated mesenteric fat stranding (Figure 4). No evidence of bowel perforation, pneumatosis intestinalis, or portal venous gas was identified. The SMA stent appeared patent on this imaging.

**Figure 5:**
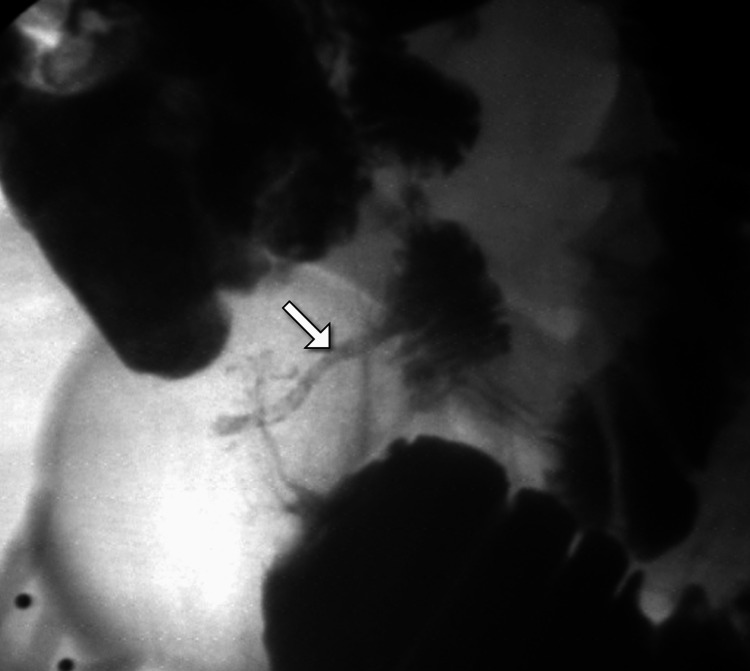
Barium small bowel study revealing terminal ileum stricture Barium follow-through study shows a segmental narrowing of the terminal ileum (arrow) with delayed passage of contrast, confirming a stricture. Findings correlate with prior imaging.

To further evaluate the degree and functional significance of the ileal narrowing, a barium follow-through study was performed. This demonstrated a fixed, short-segment stricture of the terminal ileum with delayed contrast passage and proximal mild dilatation, consistent with a chronic stricture rather than an acute inflammatory process (Figure 5). These findings, in conjunction with the patient’s history of mesenteric ischemia and recent vascular intervention, supported the diagnosis of chronic ischemic stricture of the terminal ileum, likely secondary to longstanding hypoperfusion before SMA revascularization.

The patient was managed conservatively during the acute admission with bowel rest, intravenous fluids, analgesia, and close clinical monitoring. Antiplatelet therapy was continued, and anticoagulation was not deemed necessary. Surgical consultation was obtained, and given the absence of complete obstruction, peritonitis, or bowel necrosis, a non-operative approach was initially favored. The patient’s abdominal pain gradually improved, and inflammatory markers trended downward.

At follow-up, the patient continued to experience intermittent postprandial abdominal discomfort but no signs of acute obstruction. Nutritional optimization and risk factor modification were emphasized. The patient was counseled regarding the potential need for surgical resection should obstructive symptoms worsen or fail to respond to conservative management.

## Discussion

Chronic mesenteric ischemia represents a diagnostically challenging entity due to its insidious onset, nonspecific symptomatology, and relative rarity compared with other causes of chronic abdominal pain [2,3]. While atherosclerotic disease of the mesenteric vessels is the most common underlying mechanism, clinical manifestations depend not only on the degree of arterial stenosis but also on the adequacy of collateral circulation and the duration of hypoperfusion [1-3]. The SMA plays a pivotal role in perfusing the small intestine, and significant stenosis may result in repeated episodes of subclinical ischemia that precede overt intestinal injury [2,4]. In this context, chronic ischemic damage may progress beyond functional disturbance to structural remodeling of the bowel wall, culminating in fibrosis and stricture formation.

Ischemic strictures of the small intestine are distinctly uncommon and remain underrecognized in clinical practice [1-4]. Most reported ischemic strictures involve the colon, particularly watershed areas such as the splenic flexure and rectosigmoid junction. In contrast, involvement of the terminal ileum is rare due to its typically robust collateral supply via the ileocolic and arcades [2,5]. However, prolonged or severe reduction in mesenteric blood flow, as seen in advanced SMA stenosis, may overwhelm these compensatory mechanisms [1,3]. Repeated ischemia-reperfusion injury leads to mucosal ulceration, submucosal edema, and chronic inflammation, which ultimately evolve into transmural fibrosis and fixed luminal narrowing [1-5]. This pathophysiologic process explains the delayed presentation of strictures even after successful revascularization, as irreversible bowel wall injury may have already occurred [1-5].

The present case underscores the diagnostic complexity of ischemic ileal strictures, which frequently mimic inflammatory bowel disease, particularly Crohn’s disease. Radiologic features such as segmental wall thickening, luminal narrowing, and mesenteric fat stranding are not pathognomonic and may overlap significantly with inflammatory, infectious, or neoplastic processes [2,3]. In the absence of classic Crohn’s features such as skip lesions, fistulae, or perianal disease, and given the patient’s significant vascular risk profile, an ischemic etiology became the most plausible diagnosis. Importantly, the normal lactate levels and absence of radiologic signs of acute transmural ischemia highlight that chronic ischemic injury may occur without dramatic biochemical or imaging findings, further contributing to diagnostic delay.

Endovascular revascularization has become the preferred first-line treatment for chronic mesenteric ischemia due to its minimally invasive nature and favorable short-term outcomes [6,7]. In this case, angioplasty with stent placement resulted in restoration of SMA patency and partial symptom relief, confirming the hemodynamic significance of the lesion. Nevertheless, symptom recurrence in the form of acute abdominal pain prompted further evaluation, revealing a chronic terminal ileal stricture. This temporal sequence emphasizes an important clinical principle: successful revascularization does not necessarily reverse established ischemic bowel damage. Instead, structural sequelae of prior hypoperfusion may declare themselves after blood flow has been restored, possibly due to increased functional demand on already compromised intestinal segments.

Management of ischemic small bowel strictures remains individualized and depends on symptom severity, degree of obstruction, and presence of complications [2-7]. While surgical resection is definitive for refractory or obstructive strictures, conservative management may be appropriate in selected patients without complete obstruction or peritonitis [2,6]. In the present case, non-operative management resulted in clinical improvement, allowing for close surveillance and avoidance of immediate surgery. This approach aligns with the principle of balancing symptom control against operative risk, particularly in patients with significant comorbidities. Nonetheless, clinicians should maintain a low threshold for surgical intervention if symptoms progress or nutritional compromise ensues [2,6].

## Conclusions

This case highlights chronic ischemic stricture of the terminal ileum as a rare but important sequela of longstanding mesenteric hypoperfusion, even after successful endovascular revascularization of the SMA. It underscores the need for a high index of suspicion for ischemic bowel injury in patients with significant vascular risk factors who present with ileal strictures and obstructive symptoms, particularly when imaging features are atypical for inflammatory or neoplastic disease. Timely recognition of mesenteric ischemia, appropriate vascular intervention, and continued clinical vigilance for delayed intestinal complications are essential to optimize outcomes. The key take-home message is that restoration of mesenteric blood flow may not reverse established ischemic bowel damage, and persistent or new gastrointestinal symptoms following revascularization should prompt evaluation for chronic ischemic strictures to avoid diagnostic delay and inappropriate management.
